# Tumor budding correlates with tumor invasiveness and predicts worse survival in pT1 non-muscle-invasive bladder cancer

**DOI:** 10.1038/s41598-021-97500-3

**Published:** 2021-09-09

**Authors:** Markus Eckstein, Charlotte Kimmel, Johannes Bruendl, Florian Weber, Stefan Denzinger, Michael Gierth, Maximilian Burger, Arndt Hartmann, Wolfgang Otto, Johannes Breyer

**Affiliations:** 1 Institute of Pathology, University of Erlangen-Nuremberg, Erlangen, Germany; 2 Department of Urology, University of Regensburg, Caritas St. Josef Medical Center, Landshuter Str. 65, 93053 Regensburg, Germany; 3 Institute of Pathology, University of Regensburg, Regensburg, Germany

**Keywords:** Urological cancer, Predictive markers, Prognostic markers, Urological cancer, Urology, Bladder

## Abstract

Tumor budding is defined as a single cell or a cluster of up to 5 tumor cells at the invasion front. Due to the difficulty of identifying patients at high risk for pT1 non-muscle-invasive bladder cancer (NMIBC) and the difficulties in T1 substaging, tumor budding was evaluated as a potential alternative and prognostic parameter in these patients. Tumor budding as well as growth pattern, invasion pattern and lamina propria infiltration were retrospectively evaluated in transurethral resection of the bladder (TURB) specimens from 92 patients with stage pT1 NMIBC. The presence of tumor budding correlated with multifocal tumors (*p* = 0.003), discontinuous invasion pattern (*p* = 0.039), discohesive growth pattern (*p* < 0.001) and extensive lamina propria invasion (*p* < 0.001). In Kaplan–Meier analysis, tumor budding was associated with significantly worse RFS (*p* = 0.005), PFS (*p* = 0.017) and CSS (*p* = 0.002). In patients who received BCG instillation therapy (n = 65), the absence of tumor budding was associated with improved RFS (*p* = 0.012), PFS (*p* = 0.011) and CSS (*p* = 0.022), with none of the patients suffering from progression or dying from the disease. Tumor budding is associated with a more aggressive and invasive stage of pT1 NMIBC and a worse outcome. This easy-to-assess parameter could help stratify patients into BCG therapy or early cystectomy treatment groups.

## Introduction

Urothelial carcinoma of the bladder is the 12^th^ most common malignancy worldwide^1^. Approximately 75% of patients are initially diagnosed with non-muscle-invasive bladder cancer (NMIBC)^2^. Transurethral resection of the bladder (TURB) is the initial step in the diagnosis and treatment of NMIBC^2^. Stage pT1 NMIBC is characterized by tumor infiltration of the lamina propria. Due to potential residual disease (approximately 50%) and potential understaging (approximately 10%), a second resection is recommended for all pT1 tumors within 2–6 weeks^2,3^.

Sylvester et al. recently reevaluated clinical and pathological parameters to predict disease recurrence and progression in NMIBC^4^. According to this study, the estimated progression rates were up to 44% at five years^4^. In particular, T1G3 NMIBC remains challenging since approximately 30% of patients will die from the disease, while another 30% will never suffer recurrence^5^.

This led to the investigation of additional parameters to distinguish between low aggressive and high aggressive pT1 bladder cancer. The invasion depth of the lamina propria in these tumors is a well-studied parameter. Different retrospective studies have shown a worse prognostic effect of tumor invasion beyond the muscularis mucosae or for extensive tumor invasion in the lamina propria^6–8^. A major limitation of using muscularis mucosae as the landmark is the absence of muscularis mucosae in 22% of the specimens^7^. T1 substaging is recommended in current guidelines, but the optimal method remains unclear^2^.

Another way to identify infiltrative and aggressive tumors could be by examining tumor budding. Tumor budding is defined as a single cell or a cluster of up to 5 cells at the tumor invasion front^9^. Tumor budding has been shown to be associated with adverse tumor features and worse survival in breast cancer^10–13^, squamous cell carcinoma of the lung^14^ and colorectal cancer^15,16^. In colorectal carcinoma, tumor budding has been widely investigated and shown to be prognostic in a prospective trial^16^ and its incorporation into current guidelines has been proposed^17^. In MIBC, tumor budding is associated with worse cancer-specific survival (CSS)^9^, while it seems to be associated with worse progression-free survival (PFS) in T1 NMIBC^18^.

The aim of the present study was to correlate tumor budding with tumor invasion and growth patterns in stage pT1 NMIBC and evaluate the prognostic impact of tumor budding compared to these factors and routine clinical and histopathological parameters.

## Patients and methods

### Study population

The patient cohort consisted of patients with histopathologically proven initial stage pT1 NMIBC who underwent transurethral resection of the bladder (TURB) between 2007 and 2015 at the Department of Urology of the University of Regensburg at the Caritas St. Josef Medical Center in Regensburg. All patients underwent reresection or early cystectomy, and patients with UTUC were excluded. Patients were treated according to current guidelines and the discretion of physicians. The tissue samples were stored as formalin-fixed paraffin-embedded blocks (FFPE).

All the findings, data acquisition and processing in this study comply with the ethical standards described in the latest declaration of Helsinki. The study was approved by the local ethics committee of the University of Regensburg (Nr. 16-321-101). Informed consent was obtained from all patients included in the study.

### Immunohistochemical staining for pancytokeratin and evaluation of tumor budding

Immunohistochemical (IHC) staining was performed on 4 µm tissue sections using an automated Ventana Benchmark Ultra autostainer (Ventana, Tucson, Arizona, USA). Briefly, tissue sections were deparaffinized, antigens were retrieved by heat treatment in a Tris/Borate/EDTA solution pH 8.4 (Ventana), and endogenous peroxidase was blocked with 1% H_2_O_2_. Pancytokeratin was stained using an AE1AE3-antibody cocktail (Zytomed, Friedrichshafen, Germany). The stained tissue sections were scanned using a Panoramic P250 slide scanner (3DHistech, Hungary), and tumor budding was evaluated using the open source software QuPath with a digital slide viewer^19^.

**Figure 1:**
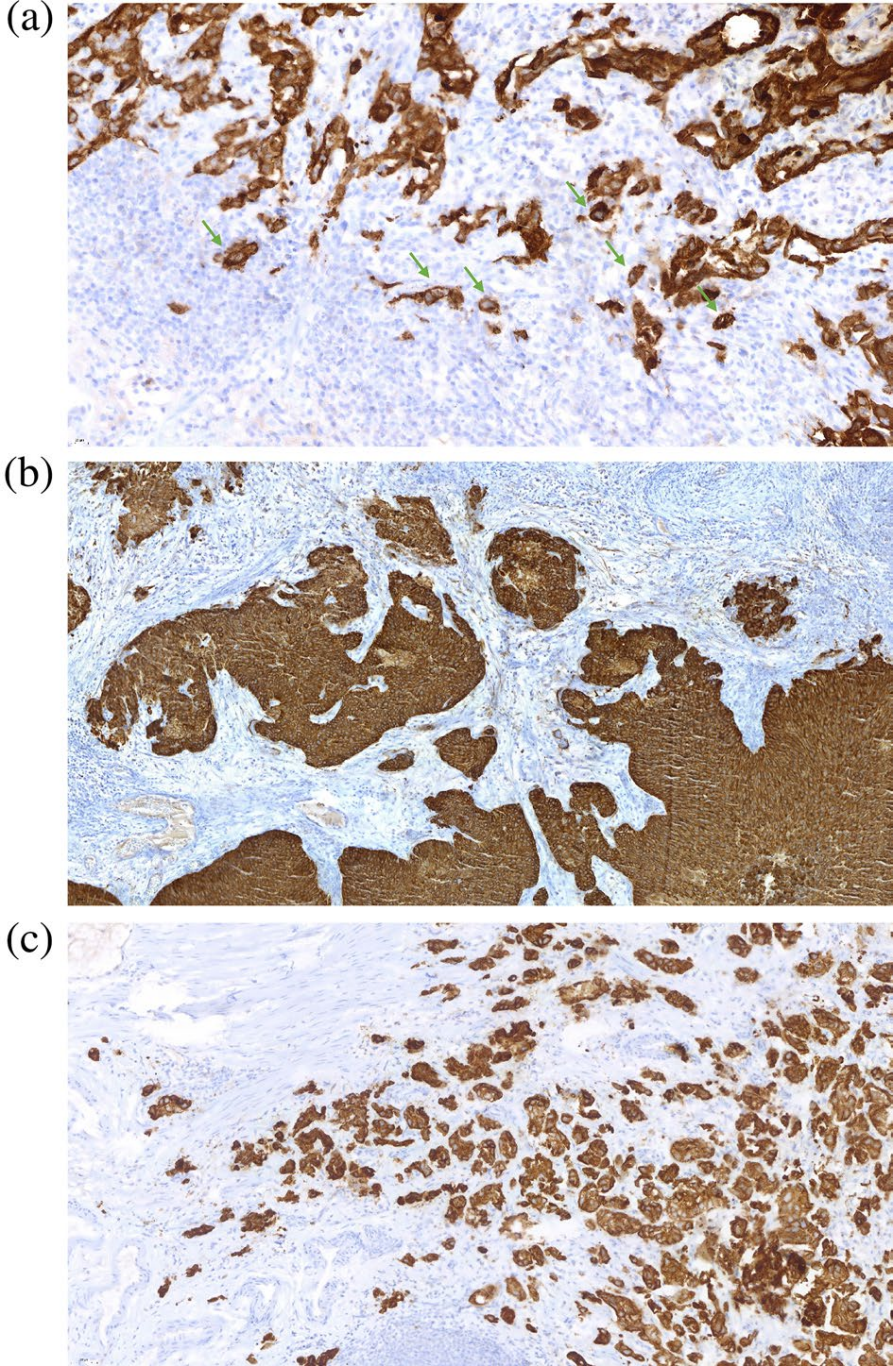
Examples of (**a**) tumor budding at the invasion front (green arrows); (**b**) cohesive growth pattern; (**c**) discohesive growth pattern.

Tumor budding was quantified in the scanned sections in 5 representative high-power fields (HPFs) under 200×magnification. Tumor buds were defined as single cells or clusters of 2–5 cells in the stroma at the invasion front of the tumor without connection to the main tumor mass. The median bud count was calculated as the median number of tumor buds in all evaluated HPFs. Evaluation of tumor budding was performed by an experienced pathologist without knowledge of the clinical characteristics and the clinical course. Figure 1a shows a representative image of tumor buds at the invasion front.

### Evaluation of tumor invasion pattern and tumor growth pattern

**Figure 2:**
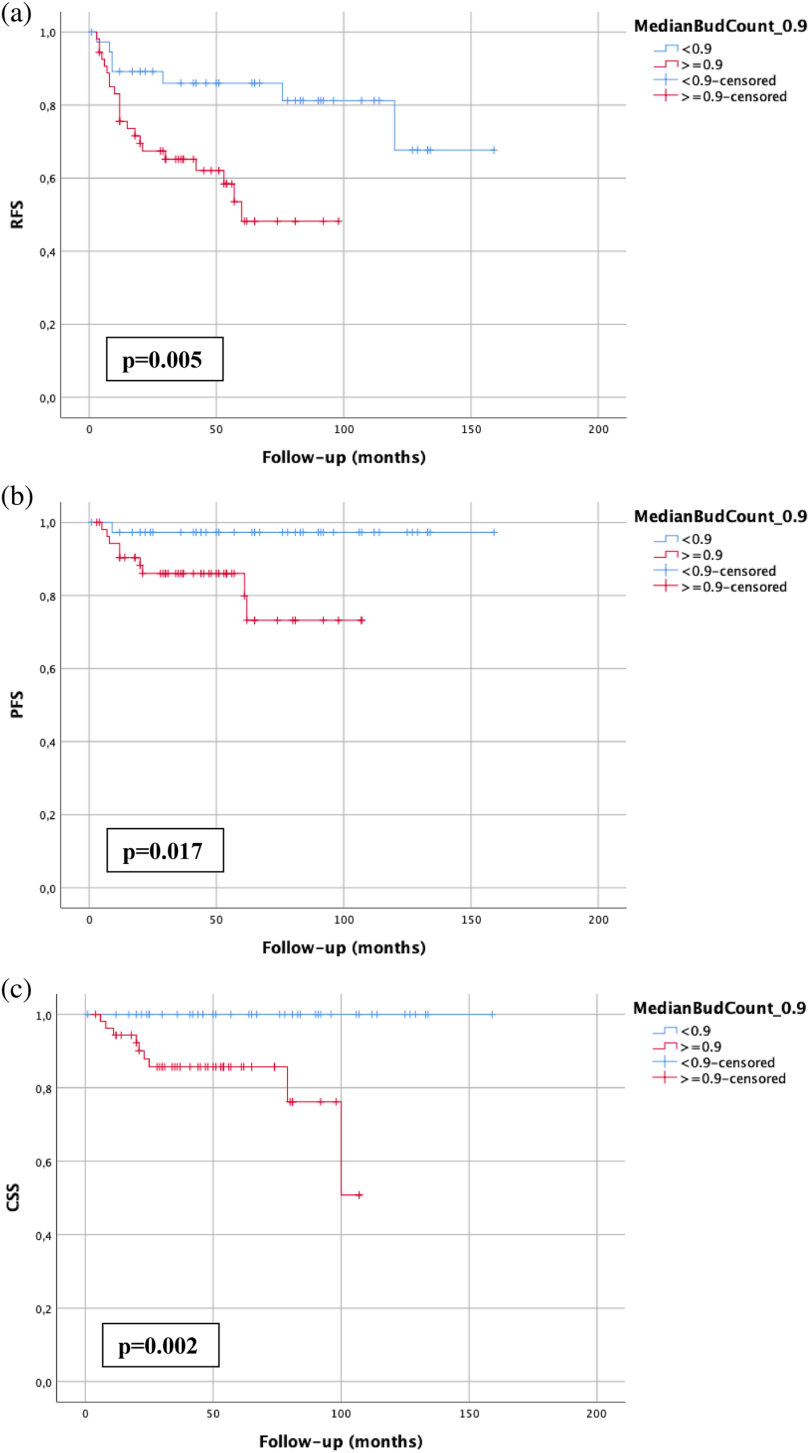
Kaplan–Meier analysis of tumor budding (cutoff: 0.9) in the total pT1 NMIBC cohort with the bladder-sparing approach (n = 92) regarding RFS (**a**), PFS (**b**) and CSS (**c**). A *p* value < 0.05 indicates significant results.

Evaluation was performed by an experienced pathologist without knowledge of the clinical characteristics and the clinical course. The growth pattern of invasive carcinoma areas was evaluated in two categories: (1) “Cohesive” with invasive tumor cells composed of large cohesive cell nests (Fig. 1b); (2) “Discohesive” with invasive tumor cells composed of small cell nests without larger cohesive areas (Fig. 1c). Invasion patterns were defined as “compact” with invasion being localized at one spot and as “discontinuous” with invasion being present in multiple spots with at least one separate invasive spot with a minimum distance of one 200 × HPF. “Limited” or “extensive” lamina propria invasion was assessed as reported previously^8^.

### Statistical analysis

Statistical analysis was performed using SPSS version 26.0 (*IBM Deutschland GmbH, Ehningen, Germany*). RFS was defined as the time from diagnosis (TURB) to the first histologically proven NMIBC recurrence (pTa, pT1 or Cis). PFS was defined as the time from diagnosis (TURB) to the first histologically proven MIBC (≥ pT2). Recurrence-free survival (RFS) rates, progression-free survival (PFS) rates and cancer-specific survival (CSS) rates were calculated by Kaplan–Meier analysis and tested for significance with the log rank test. Multivariable Cox regression analyses were used to assess the value of tumor budding, tumor invasion pattern, tumor growth pattern and clinical and histopathological parameters for RFS, PFS and CSS. The Spearman product-moment correlation coefficient r was used as a measure of the strength and direction of the linear relationship between continuous variables. Comparisons between categorical variables were performed using Fisher´s exact and chi-square tests. *P* values < 0.05 were considered statistically significant.

### Ethics approval

All the findings, data acquisition and processing in this study comply with the ethical standards described in the latest declaration of Helsinki. The study was approved by the local ethics committee of the University of Regensburg (Nr. 16-321-101).

## Results

### Patient population

Between 2007 and 2015, 141 patients were diagnosed with pT1 NMIBC in TURB. Of those, 24 had to be excluded due to insufficient tissue for IHC staining, and 5 patients were lost to follow-up. Of the remaining 112 patients, 4 had to be excluded with upstaging in TURB reresection or early cystectomy, 10 patients underwent immediate cystectomy within 4 months, and another 6 patients underwent cystectomy for NMIBC.

The remaining 92 patients who underwent the bladder-sparing approach included 76 male patients (82.6%), and the median age was 72 years. Grading according to WHO1973 was G3 in 73 patients (79.3%), concomitant carcinoma in situ (Cis) was diagnosed in 38 cases (41.3%), and 53 tumors were multifocal (57.6%). Eighty patients received instillation therapy (87%; BCG: 65 patients (70.7%), MMC: 15 patients (16.3%)). Recurrence was histologically proven in 29 patients (31.5%) and progression to MIBC in 10 patients (10.9%), with 9 dying from the disease (9.8%). Clinical and pathological parameters as well as follow-up information are displayed in Table 1.

**Table 1:** Clinical and histopathological characteristics of the pT1 NMIBC cohort with the bladder-sparing approach.

Parameter	n (%)
**Patient data**	
Total stage pT1 2007–2015	92 (100)
Female patients	16 (17.4)
Male patients	76 (82.6)
Median age (years)	72 [IQ range: 66–81]
**Clinical and pathological parameters**	
*Grading WHO1973*	
G1	0 (0)
G2	19 (20.7)
G3	73 (79.3)
*Grading WHO2016*	
Low grade	4 (4.3)
High grade	88 (95.7)
*Tumor diameter*	
< 30 mm	46 (50.0)
≥ 30 mm	46 (50.0)
*Concomitant Cis*	
Yes	38 (41.3)
No	54 (58.7)
*Focality*	
Unifocal	39 (42.4)
Multifocal	53 (57.6)
**Treatment**	
Instillation therapy	80 (87.0)
MMC	15 (16.3)
BCG	65 (70.7)
Secondary cystectomy	6 (6.5)
**Follow-up information**	
Median follow-up (months)	51 [IQ range: 28–81]
Maximum follow-up (months)	159
Recurrence ≤ pT1	29 (31.5)
Progression	10 (10.9)
Death	25 (27.2)
Death by disease	9 (9.8)

### Frequency of tumor budding and correlation with clinical and pathological parameters

ROC analysis revealed a median bud count of 0.9 as the ideal cutoff, resulting in 38 patients (41.3%) with a median bud count of < 0.9 and 54 patients (58.7%) with a median bud count ≥ 0.9. The absolute number of tumor buds in one HPF differed from 0 to 23.

Of the clinical and pathological parameters, a median bud count ≥ 0.9 was statistically significantly associated with multifocal tumors in the Chi^2^ test (*p* = 0.003).

### Correlation of tumor budding with tumor growth pattern and tumor invasion pattern

A discontinuous invasion pattern was found in 7 patients (7.6%), extensive invasion in 34 patients (37.0%) and a discohesive growth pattern in 20 patients (21.7%) (Table 2).

**Table 2:** Frequency of tumor budding, tumor invasion pattern, tumor growth pattern, and invasion.

Median Bud count (%)		Tumor invasion pattern (%)		Tumor growth pattern (%)		Tumor invasion (%)	
< 0.9	≥ 0.9	Compact	Discontinuous	Cohesive	Discohesive	Limited	Extensive
38 (41.3)	54 (58.7)	85 (92.4)	7 (7.6)	72 (78.3)	20 (21.7)	58 (63.0)	34 (37.0)

A median bud count ≥ 0.9 was statistically significantly associated with both a discontinuous invasion pattern (*p* = 0.039; Fisher`s exact test) and a discohesive growth pattern (*p* < 0.001; Fisher`s exact test) and with extensive lamina propria invasion (*p* < 0.001; Fisher`s exact test).

### Tumor budding is associated with worse RFS, PFS and CSS

In Kaplan–Meier analysis, a median bud count ≥ 0.9 was statistically significantly associated with worse RFS (*p* = 0.005), PFS (*p* = 0.017) and CSS (*p* = 0.002) (Fig. 2). Only one progression could be observed with < 0.9 median buds, and no patient with a median bud count < 0.9 died from disease. A discontinuous invasion pattern was statistically significantly associated with worse RFS (*p* = 0.001) and CSS (*p* = 0.039) but not PFS. A discohesive growth pattern (*p* < 0.001) and extensive lamina propria invasion (*p* = 0.008) were only associated with worse RFS but not with PFS and CSS. Of the clinical and pathological parameters, focality was the only statistically significant parameter (RFS: *p* = 0.014; PFS: *p* = 0.023; CSS: *p* = 0.037).

Multivariable Cox regression analysis of the best parameters in univariable analysis (recurrence: focality, tumor budding, invasion pattern, growth pattern and substaging; progression: focality, tumor budding) revealed only multifocal tumors to be statistically significant independent predictors for recurrence (OR: 2.624; 95%-CI: 1.063–6.475; *p* = 0.036). None of the parameters was predictive for progression. Since there were few events (9), multivariable Cox regression analysis for cancer-specific death was not performed.

### Tumor budding identifies a highest-risk group of pT1G3 tumors

Tumor budding with a median bud count ≥ 0.9 identified the highest-risk group of patients with pT1G3 tumors (n = 73) with worse RFS (*p* = 0.030) and CSS (*p* = 0.009) (Supplementary Fig. 1). A trend could be observed for PFS without reaching statistical significance (*p* = 0.054). Only one progression could be observed with < 0.9 median buds, and no patient with median bud count < 0.9 died from the disease.

### Absence of tumor budding predicts response due to BCG-instillation therapy

**Figure 3:**
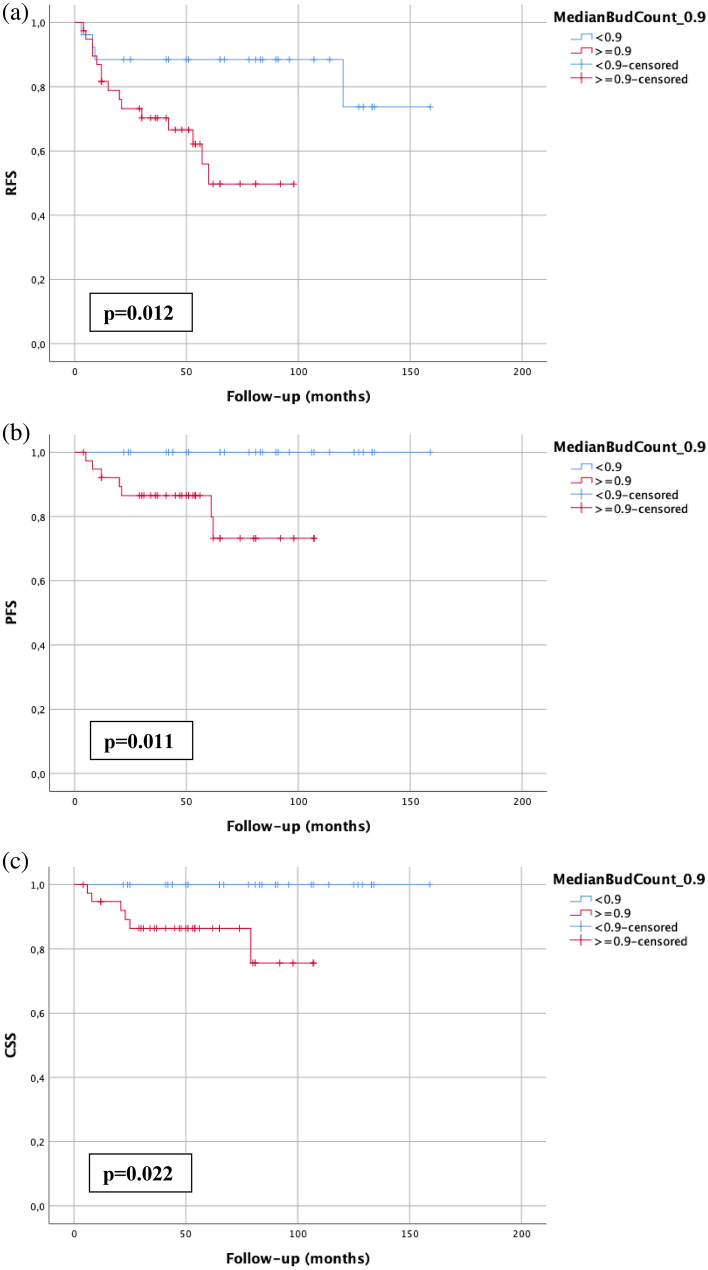
Kaplan–Meier analysis of tumor budding (cutoff: 0.9) in BCG-treated patients (n = 65) regarding RFS (**a**), PFS (**b**) and CSS (**c**). A *p* value < 0.05 indicates significant results.

Patients who received BCG instillation therapy (n = 65) showed worse RFS (*p* = 0.012), PFS (*p* = 0.011) and CSS (*p* = 0.022) when tumor budding was present (bud count ≥ 0.9) (Fig. 3). Of the patients with a median bud count < 0.9, none suffered from progression to MIBC, and none died from the disease.

## Discussion

Stage pT1 NMIBC is a challenging entity for the treating urologist. In recent years, clinical, pathological and molecular parameters have been investigated to improve the prediction of recurrence and progression and thus guide clinical decisions between radical cystectomy and bladder-sparing therapy with BCG instillations. Clinical and pathological parameters have been implemented in current guidelines, resulting in all stage T1 tumors being assessed as high-risk disease^2,4^.

To stratify stage T1 tumors according to their invasiveness and aggressiveness, different approaches to T1 substaging have been described and investigated^6–8^. The study cohort in the present retrospective study was composed of patients with a bladder-sparing approach to eliminate a potential bias regarding cystectomy for NMIBC. Tumor budding was compared to tumor invasion (limited vs. extensive^8^), tumor invasion pattern (compact vs. discontinuous) and tumor growth pattern (cohesive vs. discohesive). Tumor budding was associated with extensive lamina propria invasion, a discontinuous invasion pattern and a discohesive tumor growth pattern displaying an aggressive and invasive T1 phenotype. Furthermore, tumor budding was associated with multifocal tumors, which was the only clinical and histopathological parameter associated with worse outcome, and this result might be due to the relatively small sample size and the pure T1 cohort. A correlation between tumor budding and adverse clinical and pathological parameters were also found in breast cancer and squamous cell carcinoma of the lung^13,14^.

Tumor budding assessment with median bud count in 5 HPFs using pancytokeratin staining was easy and reproducible according to two independent reviewers. AE1/AE3 has been used to determine tumor budding in MIBC^9^, squamous cell carcinoma of the lung^14^ and colorectal carcinoma^20^. Horcic et al. and Karamitopoulou et al. showed that performing pancytokeratin staining with AE1/AE3 in colorectal carcinoma and calculating tumor budding in HPFs was accurate and reproducible with high interobserver agreement^20,21^.

Tumor budding was associated with worse RFS, PFS and CSS, with only one progression and no patient dying from disease without tumor budding (median bud count < 0.9). Tumor budding is an already established parameter for worse RFS and CSS survival in colorectal carcinoma^15–17^. In squamous cell carcinoma of the lung, worse overall survival, CSS and PFS could be observed when tumor budding was present^14^. Soriano et al. showed in 106 patients with MIBC that tumor budding was associated with worse CSS^9^. In a study with T1 NMIBC, Fukumoto et al. showed a worse PFS regarding tumor budding^18^, which is in line with our results. However, in a study with 60 patients with MIBC, no association between tumor budding and PFS and OS was observed^22^. This may be due to the small sample size and the high rates of tumor budding in this study.

Furthermore, the cutoff of 0.9, indicating a worse prognosis in patients with tumor budding in the present study, seems to be an easy and rapidly assessable parameter in routine clinical practice. Soriano et al. determined 14 tumor buds as the cutoff in their study in MIBC^9^. Garfinkle et al. described a cutoff of 10 tumor buds per HPF in their study in colorectal carcinoma^15^. Compared to this, the cutoff of one (0.9) tumor bud would be easier to assess; however, this has to be evaluated and proven in a multicenter prospective setting.

T1 substaging is recommended in the current EAU guidelines, but the ideal method to determine T1 substaging remains unknown^2^. Recently, metric substaging (limited vs. extensive lamina propria invasion) was compared to T1 substaging according to muscularis mucosae invasion in 601 patients with pT1 NMIC^7^. In this multicenter retrospective study, only metric substaging was associated with worse PFS and CSS ^7^. Muscularis mucosae was not detected in 22% of the specimens, limiting substaging using this method^7^. Previously, Bertz et al. showed that metric substaging is better than using muscularis mucosae invasion^8^. In our study, tumor budding correlated with tumor invasion in a statistically significant manner, and it was the only parameter that showed significant differences regarding RFS, PFS and CSS and was independent of the resection depth.

The specimens used in the study were mainly from fractioned TURB samples, and smaller tumors were resected en bloc. En bloc TURB has been shown to be associated with improved pathological and oncological outcomes^23^. En bloc resection might improve the substaging of T1 NMIBC and the assessment of tumor budding. Tumor budding in the fractioned specimens was easy to assess. However, it would be worth investigating this in a prospective setting with documented resection techniques.

Furthermore, in the present study, tumor budding assessment identified a subgroup of highest-risk patients with pT1G3 tumors with worse RFS and CSS. Moreover, in those patients treated with BCG instillations, the absence of tumor budding was associated with an excellent response to this treatment with no progression and no patient dying from the disease. Thus, tumor budding could be used to stratify patients into BCG instillation therapy (no tumor budding) and early cystectomy (presence of tumor budding) at the initial diagnosis of stage pT1 NMIBC. As BCG shortage has been a problem in routine practice from time to time^24^ and due to its toxic side effects leading to discontinuation of BCG, this stratification would help identify those patients who would benefit this treatment. Furthermore, it has been shown that patients with high-risk NMIBC who suffer from progression to MIBC show worse survival than patients^25^, highlighting the need to identify those patients at initial diagnosis. However, the high morbidity and 90-day mortality of up to 9% of radical cystectomy have to be taken into account^26^.

### Limitations

Data assessment was performed in a retrospective single-center setting with a small sample size. Therefore, a multicenter and prospective study with a larger cohort is essential, especially for addressing further questions such as the impact of the TURB resection technique (fractioned vs. en bloc) on the outcome and assessment of substaging and tumor budding. Furthermore, evaluation of tumor budding might be prone to interobserver variability, which should be evaluated in a multicenter setting.

## Conclusions

Tumor budding is associated with more aggressive and invasive tumors in stage pT1 NMIBC and is associated with worse outcomes in those patients. Furthermore, a lack of tumor budding predicts the response to BCG treatment. Following validation in a large multicenter prospective cohort, the implementation of this easy-to-assess parameter could help stratify pT1 patients into those who should receive BCG instillation therapy and those who should be followed up more intensely or should receive early cystectomy.

## Supplementary Information

Supplementary Figure S1.

## Data Availability

The datasets generated and/or analyzed during the current study are available from the corresponding author upon reasonable request.
