# Supplementary material for: Tumor budding correlates with tumor invasiveness and predicts worse survival in pT1 non-muscle-invasive bladder cancer
Source: Sci Rep. 2021 Sep 9;11:17981. doi: 10.1038/s41598-021-97500-3 (PMC8429693; doi:10.1038/s41598-021-97500-3)
Supplement: Supplementary file 1 — Supplementary Figure S1. [file 41598_2021_97500_MOESM1_ESM.docx]

**Suppl. Fig. 1:** Kaplan-Meier-analysis of tumor budding (cut-off: 0.9) in the T1G3 tumors (n=73) regarding RFS (a) and CSS (b). p-value<0.05 indicates significant results. Figures drawn with SPSS 26.0 (*IBM Deutschland GmbH, Ehningen, Germany*).

a)


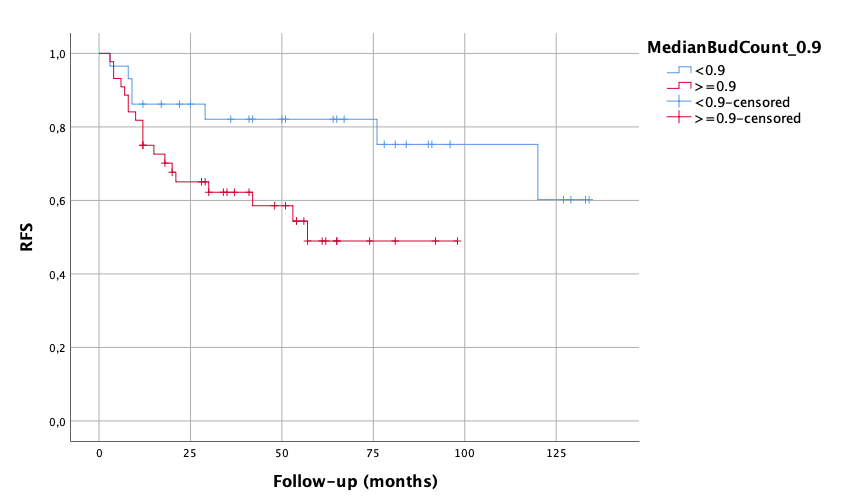


**p=0.030**

b)


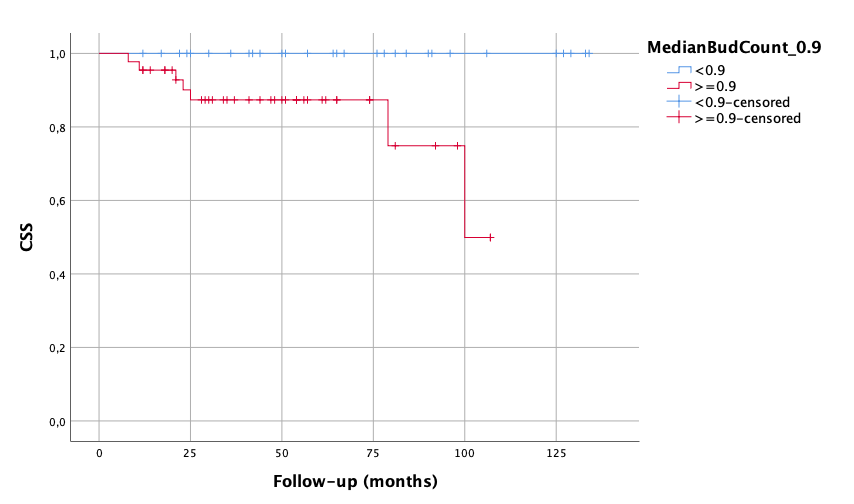


**p=0.009**
